# Determination of Prestress Losses in Existing Pre-Tensioned Structures Using Bayesian Approach

**DOI:** 10.3390/ma15103548

**Published:** 2022-05-16

**Authors:** Martin Moravčík, Jakub Kraľovanec

**Affiliations:** Department of Structures and Bridges, Faculty of Civil Engineering, University of Zilina, Univerzitna 8215/1, 010 26 Zilina, Slovakia; martin.moravcik@uniza.sk

**Keywords:** Bayesian approach, prestressing force, saw-cut method, assessment, pre-tensioned members

## Abstract

Deterioration of materials and structures is an unavoidable fact, and prestressed concrete structures are not an exception. The evaluation of load-carrying capacity and remaining service life includes collecting various information. However, one type of information is essential and the most important, the state of prestressing, which inevitably decreases over time. Currently, many possible methods for the evaluation of prestressing are available. These techniques are part of the structural assessment and provide residual prestressing force value which is later used in the evaluation process. Therefore, it is suitable to provide the value of prestressing force based on certain probabilistic backgrounds. This study addresses the determination of residual prestressing force in pre-tensioned railway sleepers one year after their production, using the so-called Bayesian approach. This technique is focused on the validation of results obtained from the application of the non-destructive indirect saw-cut method. The Bayesian approach considers analytic calculation as the primary method of prestressing determination. In this paper, Monte Carlo simulation was used to determine the total variability that defines all Bayesian systems of probability functions. Specifically, a total of 1000 simulations was applied, and the current random vector of prestressing force derived from the analytical calculation has been assumed as a normally distributed function. Finally, obtained results for different depths of saw-cuts are compared. The results of the experimental and statistical determination of residual prestressing force provide its value with a 95% confidence level. This study suggests that the implementation of the probability approach can be an effective tool for determining prestress losses.

## 1. Introduction

When assessing existing prestressed concrete structures, the determination of the residual prestressing force is an essential and inevitable task [1,2]. Prestressing force value obviously decreases over time and its determination should consider all potential prestress losses [3,4]. However, acquiring crucial knowledge about the exact value of prestressing force acting on the structure is quite difficult. Of course, for reliable assessment additional information considering the structure’s condition needs to be obtained [5,6]; it is possible to collect this data using common testing methods [7]. This general knowledge includes many material properties, such as information about the real geometry of structural members and reinforcement, damage and deterioration (obtained from visual inspections); data containing the effect of significant overloading of the structure, etc. [8,9].

Generally, the analytical or numerical calculation of prestressing force value is the standard approach. Therefore, required input data consists of the age of the structure, its geometry, reinforcement parameters, and layout. Moreover, necessary material properties can be obtained using a wide range of standard material testing procedures [10,11,12]. However, all collected data that affect the prestressing force have a natural strongly stochastic character, especially due to effects such as the rheology of concrete (creep and shrinkage) or steel relaxation [13].

In Bayesian philosophy, the analytic calculation (the primary method of prestress determination) can be considered as the prior hypothesis, together with its probability. Nevertheless, other new or additional relevant information can also be desirable and useful, especially regarding the unknown value of a prestressing force that is acting on the structure [14,15,16]. Likewise, several indirect techniques or structural tests can be used, such as the saw-cut method or structural response method [17,18,19]. These methods are based on observation of the structural behavior after the application of a known load. In the case of the saw-cut method, normal stress (strain) relief is observed. On the other hand, the structural response method evaluates prestressing based on the measurement of deflection, strain (normal stress) change or width of crack resulting from the external load [20,21]. All new relevant information can be taken into account and combined with the prior probabilistic model using updated techniques. These results are so-called posterior probabilistic models, which may be used to obtain an enhanced assessment of current prestressing force.

Updating the probability distribution of a basic variable is commonly based on the Bayesian approach described briefly below. Two individual events, A and B, are studied. The conditional probability P (A|B) of event A, given event B has occurred with a non-zero probability P (B), is defined as:

P (A|B) = P (A ∩ B)/P (B) = [P (A) × P (B|A)]/P (B)(1)

where P (A|B) is a conditional probability, i.e., the probability of event A occurring given that B is true. It is also called the posterior probability of A given B. P (B|A) is also a conditional probability, i.e., the probability of event B occurring given that A is true. It can also be interpreted as the likelihood of A, given a fixed B, because P (B|A) = L (A|B) × P (A) and P (B) are the probabilities of observing A and B, respectively, without any given conditions; they are known as the marginal probability or prior probability. A and B must be different events.

**Figure 1:**
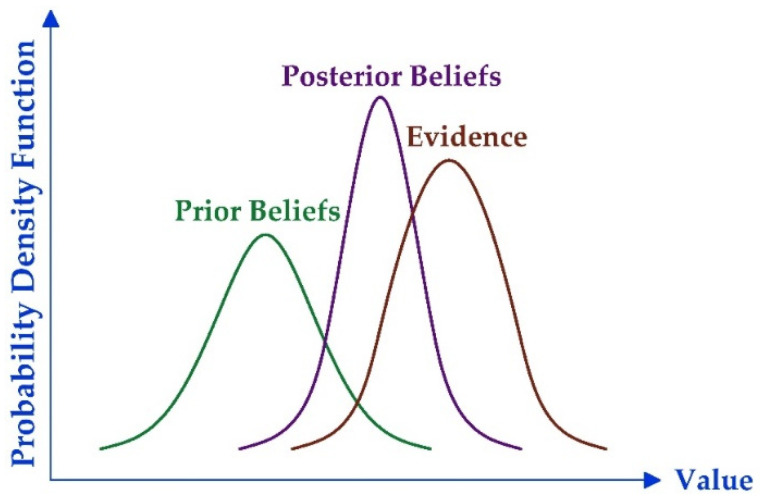
Bayesian concept.

The Bayesian theorem can also be applied to the hypothesis verification tool, and graphically interpreted using probability density functions as presented in Figure 1.

In fact, the input data are random variables, thus the probability distribution function can be used to represent the data {x_1_, x_2_,…, x_n_} with distribution parameters of θ. The concept of conjugacy in Bayesian statistics is used. Conjugacy occurs if the posterior distribution is in the same family of probability density functions as the prior belief, but with new parameter values. These values are updated to reflect what has been understood from the obtained data. Bayes’ theorem, expressed in terms of probability distributions, appears as:

f (θ|data) = [f (θ) × f (data|θ)]/[∫ f (data|θ) × f (θ) × dθ](2)

where f (θ|data) is the posterior distribution for the parameter θ; f (data|θ) is the sampling density for the data, which is proportional to the likelihood function, only differing by a constant that makes it a proper density function; f (θ) is the prior distribution for the parameter θ; and f (data) is the marginal probability, f (data) = ∫ f (data|θ) × f (θ) × dθ.

A number of closed-form solutions for Equation (2) can be found for special types of probability distribution functions known as the natural conjugate distributions. In cases where no analytical solution is available, first order reliability method (FORM)/second order reliability method (SORM) techniques can be used to assess the posterior distribution. If the normal–normal conjugate family N (μ,σ^2^) is taken into account, Bayes’ theorem leads to the posterior distribution for μ and σ^2^ given the observed data to take the form:

p (μ,σ^2^|x_1_, x_2_,…, x_n_) = [p (μ,σ^2^) × p (x_1_, x_2_,…, x_n_|μ,σ^2^)]/Normalizing Constant (3)

where p (μ,σ^2^) is the joint prior distribution function. The likelihood function for (μ,σ^2^) is proportional to the sampling distribution of the data, L (μ,σ^2^) ∝ p (x_1_, x_2_,…, x_n_|μ,σ^2^), so that the posterior distribution can be re-expressed in proportional form. The symbol ∝ means “proportional to“. According to the Joint Committee on Structural Safety (JCSS) Probabilistic Model Code described in [14] and [22], Equation (3) can be expressed for normal distribution in engineering-acceptable form for μ and σ, given as:

f′(μ,σ) = k × σ ^− [δ(n′) + v′ + 1]^ exp {−[(1/2 × σ^2^)] × [v′ × (s′)^2^ + n′ × (μ − m′)^2^]} (4)

where k is the normalizing constant; δ (n′) = 0 for n′ = 0; δ (n′) = 1 for n′ > 0; m′ is the sample mean; s’ is the sample standard deviation; n is the sample size; and v′ = n − 1 is the number of degrees of freedom. Then, the predictive value of {X} can be found from:

{X} = m″ + t_v’’_ × s″ × {1 + 1/n″} ^0.5^ (5)

where t_v″_ has a central Student‘s t-distribution.

The present study is based on results from the application of the non-destructive saw-cut method performed on pre-tensioned members—e.g., railway sleepers. The method aims to isolate concrete block from acting forces by means of saw-cuts. Residual prestressing force is subsequently calculated from normal stress relief initiated by sawing [17,18]. The ratio of isolation of concrete block is dependent on the parameters of saw-cuts—depth and axial distance [23]. One of the main advantages of this technique is that it has negligible local impact on the prestressed concrete structure. When using the saw-cut method on an unloaded structure, the prestressing force value is easy to calculate because the determination of normal stress resulting from the dead load is obvious. However, if the investigated member is also loaded by an external load, additional normal must be taken into account [20,21].

## 2. Analytical Calculation of Prestressing Force Value

**Figure 2:**
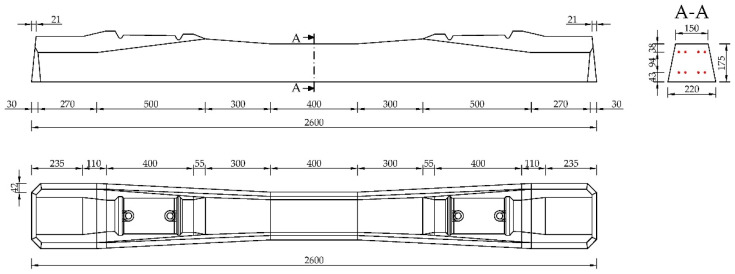
Analyzed prestressed concrete sleeper B70 W-49G.

The analysis was performed on a prestressed concrete sleeper, which is one of the standard pre-tensioned members produced in the manufacturing process. In particular, our specimen is sleeper type B70 W-49G, as illustrated in Figure 2.

Declared basic parameters entered into the analytical calculation of prestressing force P_m_ (t) (kN) are listed in Table 1.

The prestressed concrete sleeper was designed from concrete class C50/60 [24]. Therefore, the modulus of elasticity, E_cm_ = 37,000 MPa, was assumed. Calculation of prestressing force P_m_ (t) in time t = 365 days was performed according to the Eurocode 2—Slovak national implementation STN EN 1992-1-1 [25]. Prestressing of the sleeper was provided by eight wires with a smooth surface and a diameter of 7 mm. The initial prestressing force was derived from Equation (6) considering the value of initial stress in the wire, σ_p,in_ = 1380 MPa. The bending moment due to self-weight is M_Go_ = 0.798 kNm.

σ_pm,max_ = min [0.80 × f_pk_; 0.90 × f_p,0.1k_] (6)

Long-term prestress losses have the highest influence on the prestressing force value’s decrease over time. They were determined using standard Equations (7) and (8). Prestress losses due to steel relaxation are:

Δσ_p,r_ (t,t_0_) = −σ_pi_ × k_1_ × ρ_1000_ [%] × 10^−5^ × e ^μ × k2^ × [t/1000]^0.75 × (1 − μ)^ = −169.3 MPa (7)

where k_1_ = 5.39; k_2_ = 6.7; ρ_1000_ = 8.0; μ = 0.79.

Δσ_p,r+s+c_ = −{0.8 × Δσ_p,r_ (t,t_0_) + ε_cs_ (t,t_0_) × E_p_ + (E_p_/E_cm_) × φ (t,t_0_) × σ_c,(Pm0+G0)_ (t,t_0_)}/{1 + (E_p_/E_cm_) × (A_p_/A_c_) × [1 + (A_c_/I_c_) × e_p_^2^] × [1 + 0.8 × φ (t,t_0_)} = −310.3 MPa (8)

where ε_cs_ (t,t_0_) = 5.01; and φ (t,t_0_) = 1.69.

Corresponding prestressing force, considering the prestress losses after 365 days in one prestressing wire, is P_m_ (t) = 41.2 kN.

All analytical systems contain a certain degree of variability. When these systems are formed by a combination of random variables, the resulting variability of the system generally cannot be found in a closed-form approach. An alternative approach that allows the estimation of variability in a system, given the variability of its components, is Monte Carlo simulation (MCS). In this study, MCS was used to determine the total variability that defines all Bayesian systems of probability functions. In our case, a total of 1000 simulations were applied.

**Figure 3:**
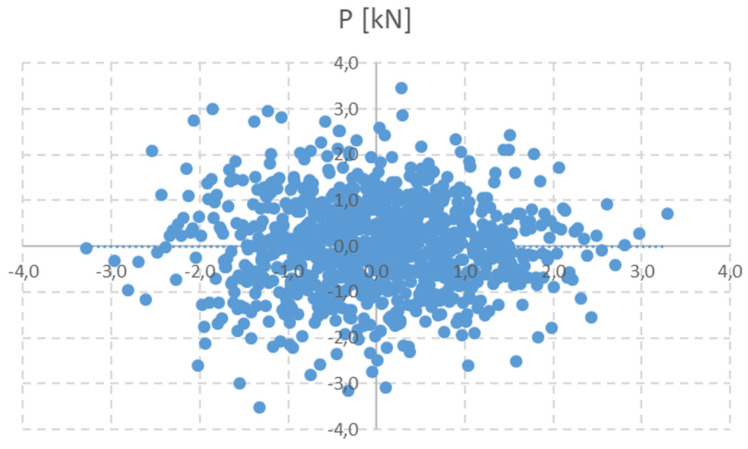
Normally distributed values of P_calc_.

**Figure 4:**
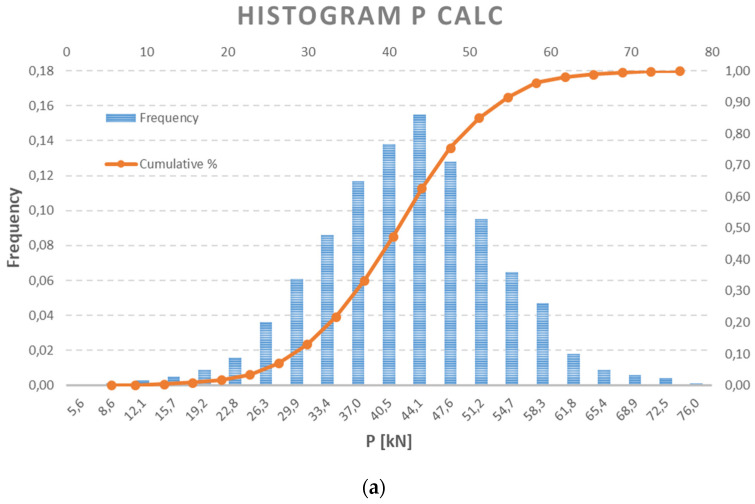

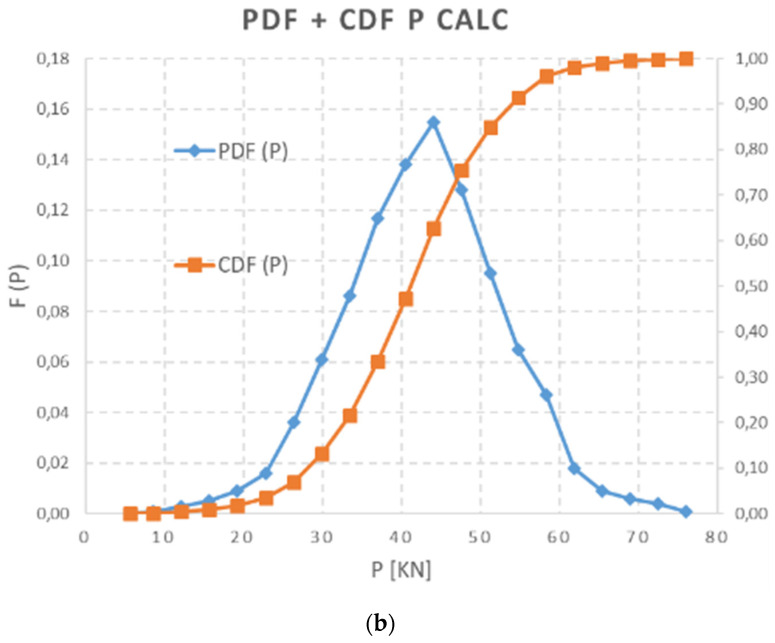
(**a**) Histogram and CDF of P_calc_; and (**b**) PDF and CDF of P_calc_.

The current random vector of prestressing force {P_calc_} derived from the analytical calculation has been assumed as a normally distributed function, as in [15], based on a random generation with a known mean value of 40.96 kN and a standard deviation of 10.20 kN. In the first approximation, this level corresponds to the estimated variation coefficient of 25%. Of course, it is appropriate to have all components in Equation (7) or (8) as a random variable. Specifically, they have mean values according to Table 1, and the strength and modulus of elasticity parameters have a common coefficient of variation (CV), CV = 5%, and for the cross-sectional parameters, CV = 3%. However, in this simulation, the consequence of the central limiting theorem was applied. Estimated prestress force is presented using a histogram, probability density function (PDF) and cumulative distribution function (CDF) in Figure 3 and Figure 4. It can be considered as the joint prior probability function, as in Equation (3). Statistical parameters are listed in Table 2.

## 3. Experimental Program—Saw-Cut Method

**Figure 5:**
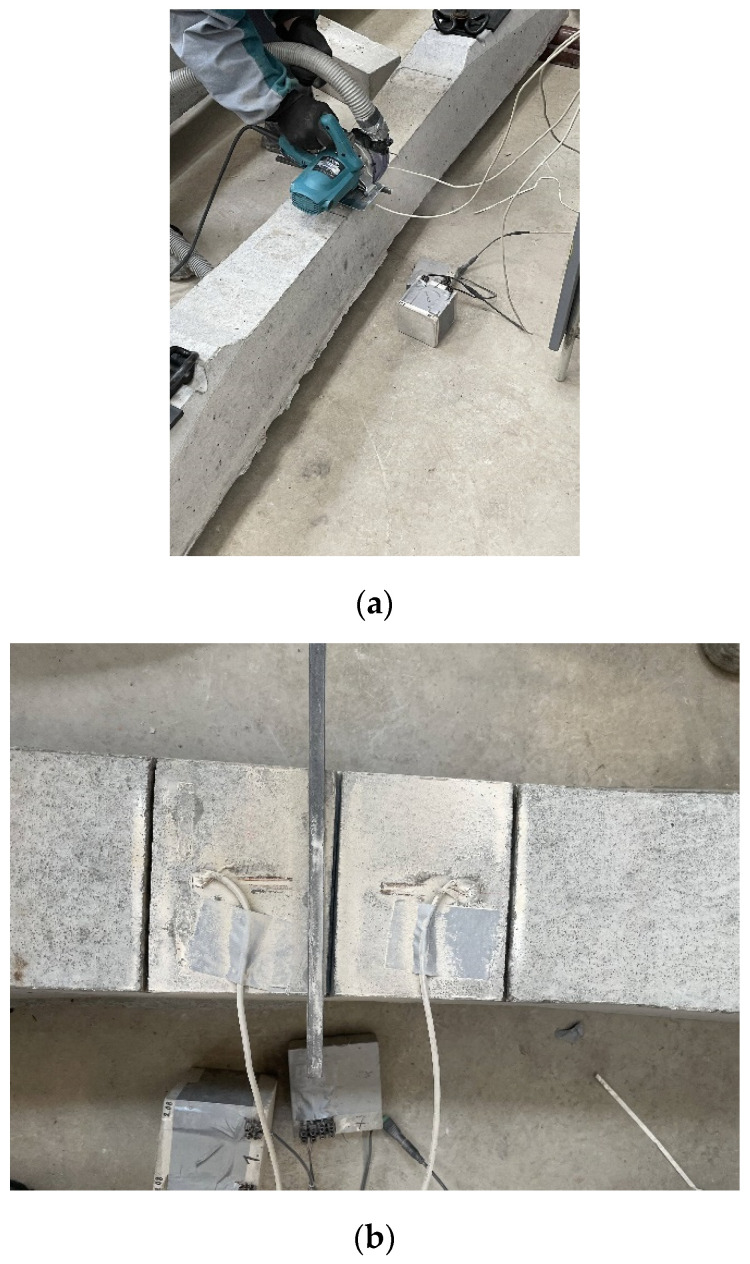
The measurement: (**a**) application of saw-cuts and (**b**) view of saw-cuts.

**Figure 6:**
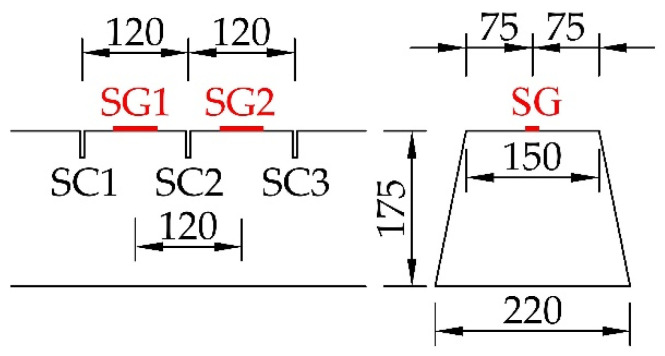
Mid-span area: position of saw-cuts (SC) and strain gauges (SG).

The application of the saw-cut method on prestressed concrete sleepers consisted of three saw-cuts. Their axial distance was 120 mm, and sawing was performed gradually (depths of 10, 20 and 30 mm). The maximal depth of saw-cuts was chosen with regard to the layout of prestressing wires in the sleepers, as we intended to avoid cutting them and affecting the structural integrity of pre-tensioned members. For the experiment, the upper edge of the specimen with a straight and smooth surface in the mid-span area was chosen. This location provided suitable conditions for the installation of strain gauges and subsequent measurement of strain release after sawing. The measurement is presented in Figure 5. For strain recording, linear foil strain gauges HBM LY41-50/120 made of ferritic steel (temperature matching code “1”: 10.8 × 10^−6^/K) with a measuring grid length of 50.0 mm and a total length of 63.6 mm were installed. The position of strain gauges can be seen in Figure 6. The prestressed concrete sleepers were supported by two lines at a distance of 0.1 m from the ends. Supports were provided using so-called steel rollers; as a consequence, the specimens behaved as simply supported beams with an effective length of 2.4 m.

All the equations and assumptions mentioned are based on the linear distribution of normal stress which can be assumed in the case of the uncracked prestressed concrete structure. Therefore, in the case of an already pre-cracked structure, such an assumption should not be considered. After sawing, some local nonlinearities could be observed, but the area adjacent to installed strain gauges should not be significantly influenced given an axial distance of 120 mm.

**Figure 7:**
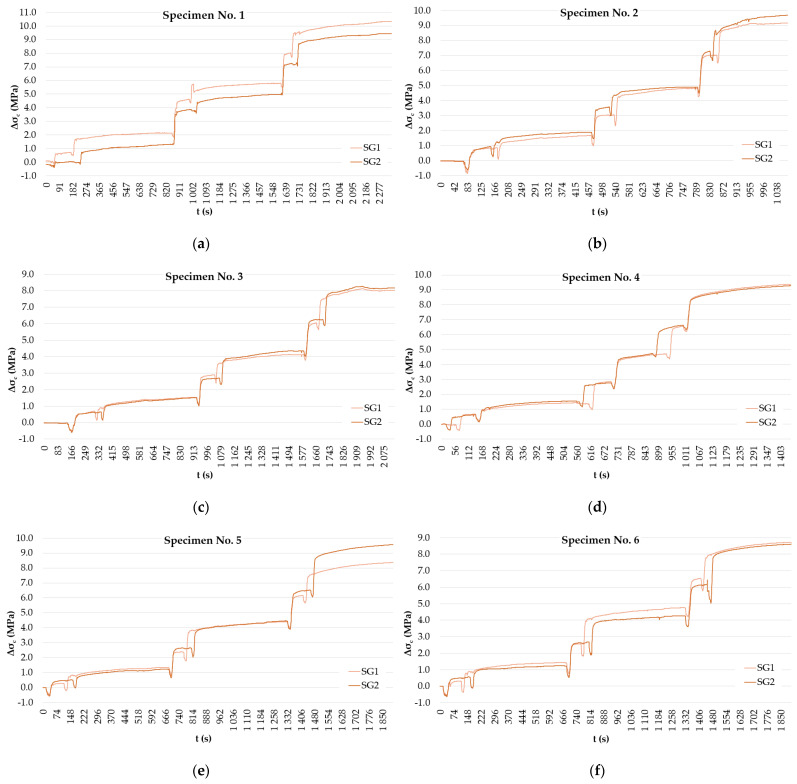
Normal stress readings on strain gauges after the application of saw-cuts. Specimens—1 (**a**); 2 (**b**); 3 (**c**); 4 (**d**); 5 (**e**); 6 (**f**).

Normal stress readings from the measurement are displayed in Figure 7 and listed in Table 3. Evaluation of obtained results was based on the real value of the concrete’s modulus of elasticity, which was determined using removed cylindrical samples (37.4 GPa). The real modulus of elasticity value was in compliance with Eurocode 2 [25].

**Figure 8:**
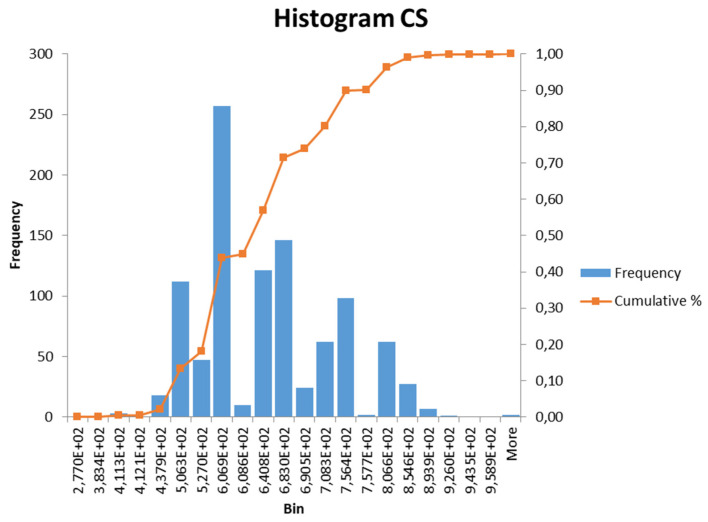
Histogram and CDF of CS parameter.

Depending on the depth of a saw-cut, experimentally determined prestressing force value P_exp,i_ can be calculated according to Equation (9):

P_exp,i_ = {k_i_ × Δσ_c,I_ − [(M_G0_ × z_upp_)/I_c_]}/CS (9)

where k_i_ is the “calibration factor of the depth of saw-cut” determined according to a parametric study based on nonlinear numerical simulations. This constant represents the ratio between released normal stress after the application of saw-cuts and initial normal stress in a prestressed concrete member. The deeper a saw-cut is in the shorter axial distance we choose, the more normal stress is released, and the calibration factor has a lower value. More information about the calibration factor and its determination can be found in [23]. Δσ_c,i_ is the released normal stress value dependent on the depth of a saw-cut, and CS is a cross-sectional function, as in Equation (10):

CS = −(8/A_c_) − [(−4 × e_p,bott_ + 4 × e_p,upp_ × z_upp_)/I_c_] (10)

where e_p,bott_ is the distance from the cross-section center to the center of the bottom wires; and e_p,upp_ is the distance from the cross-section center to the center of the upper wires. All cross-sectional parameters used in Equation (10) were considered as random variables according to Table 4. A histogram and CDF of the cross-sectional function CS can be seen in Figure 8.

## 4. Prestressing Force Distribution Using Bayesian Approach

**Figure 9:**
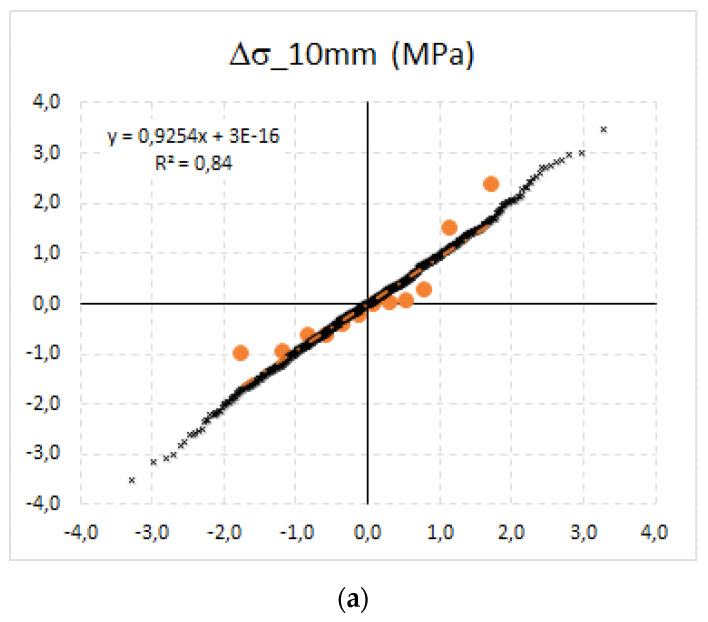

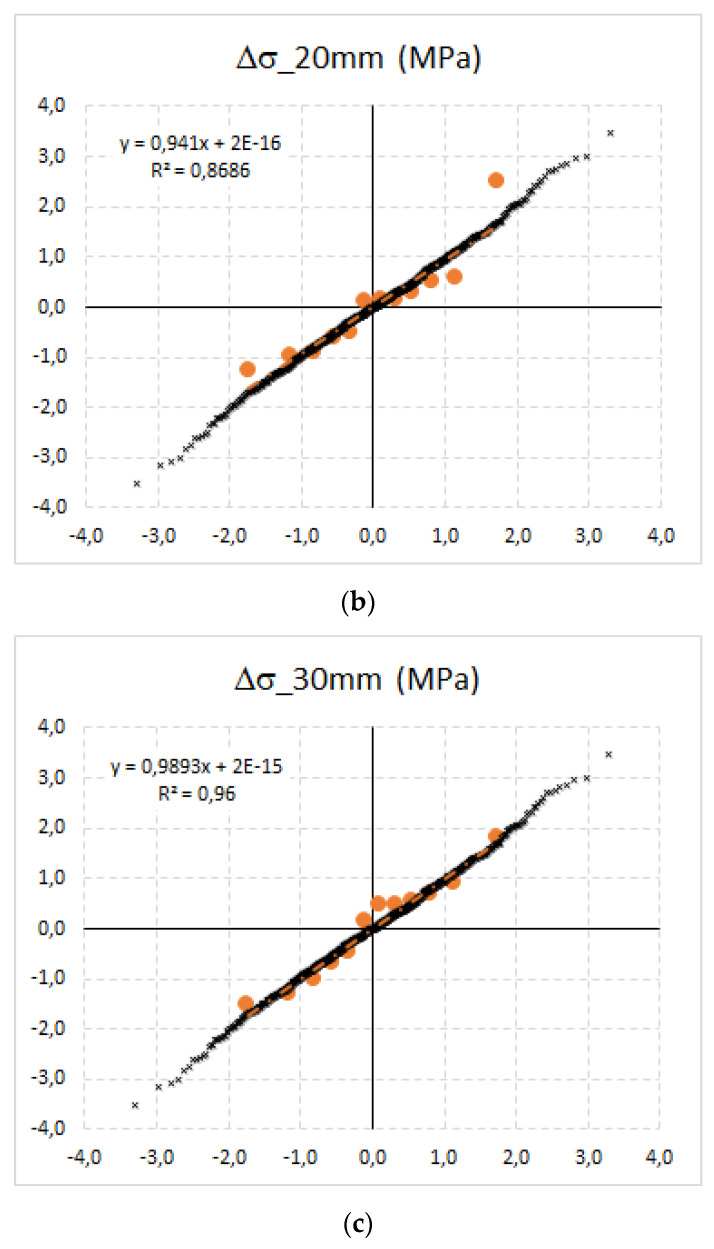
Q-Q-plot tests for released normal stress (MPa): (**a**) Δσ_c,10_; (**b**) Δσ_c,20_; and (**c**) Δσ_c,30_.

Measured released stresses {Δσ_c,10_; Δσ_c,20_; and Δσ_c,30_} in prestressed concrete sleepers were tested using the Q-Q-plot method as one of the principal testing methods, and approximated using normally distributed data that were randomly generated using the MC simulations. Additionally, over 1000 simulations were performed. The results of Q-Q-plot testing can be seen in Figure 9. The data with the best agreement with the linear regression parameter R^2^ = 0.96 were gained from the 30 mm deep saw-cuts, in which the highest stress relief was reached (approximately 72% of total assumed initial normal stress). This corresponds with the calibration factor k_30/120_ = 1.39. On the other hand, a 10 mm deep saw-cut released only 12% of initial normal stress. For this depth, the calibration factor is k_10/120_ = 8.40.

**Figure 10:**
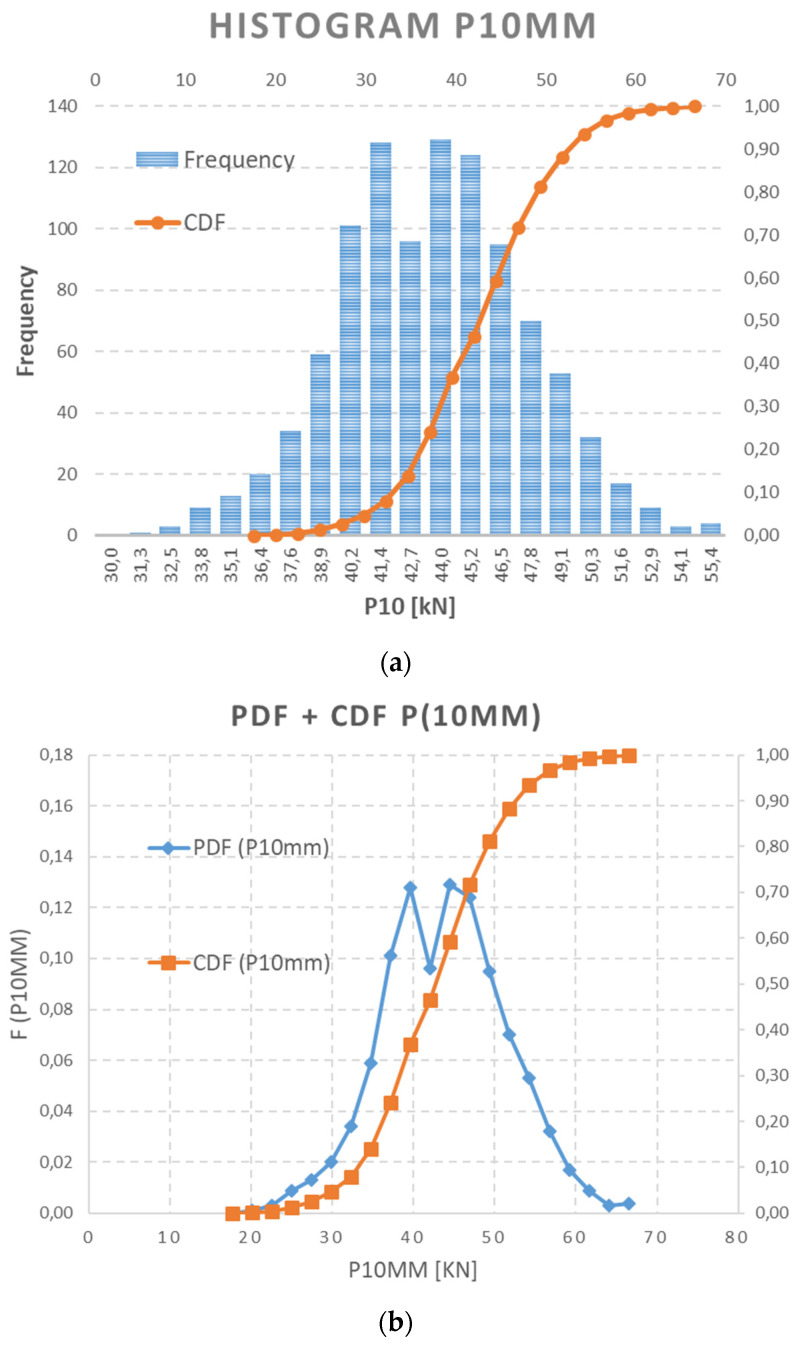
Δσ_c,10_—(**a**) histogram of generated data and (**b**) PDF and CDF.

**Figure 11:**
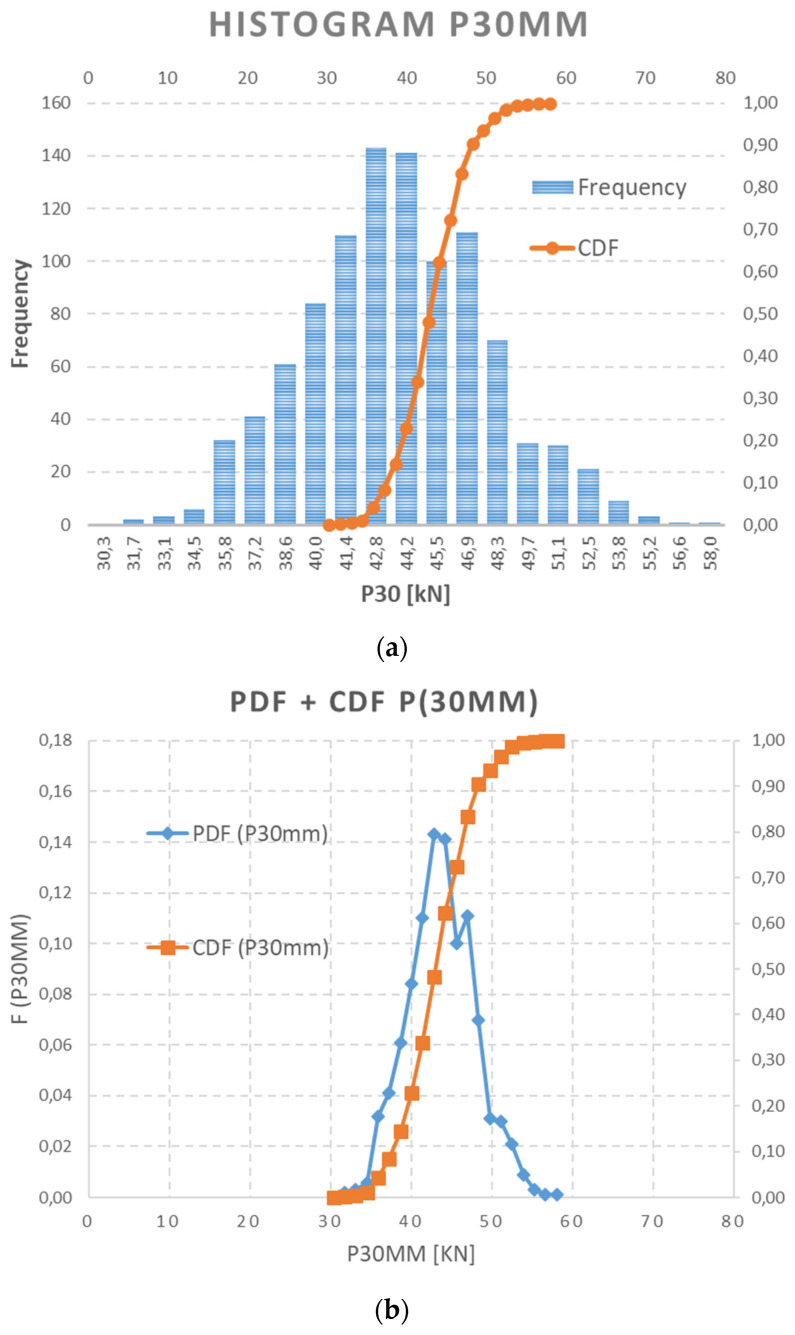
Δσ_c,30_—(**a**) histogram of generated data and (**b**) PDF and CDF.

The random vector of prestressing force {P (imm)} = {P (10 mm); P (20 mm); P (30 mm)} based on a measured data set of released normal stress is assumed according to Equation (9). In this paper, only worst fitted data (a saw-cut depth of 10 mm) and best-fitted data (a saw-cut depth of 30 mm) were chosen for graphical interpretation on histograms. PD and CD functions are illustrated in Figure 10 and Figure 11. Consequently, some differences between the functions P (10 mm) and P (30 mm) are obvious from the basic statistical parameters of both of chosen data sets, as listed in Table 5.

**Figure 12:**
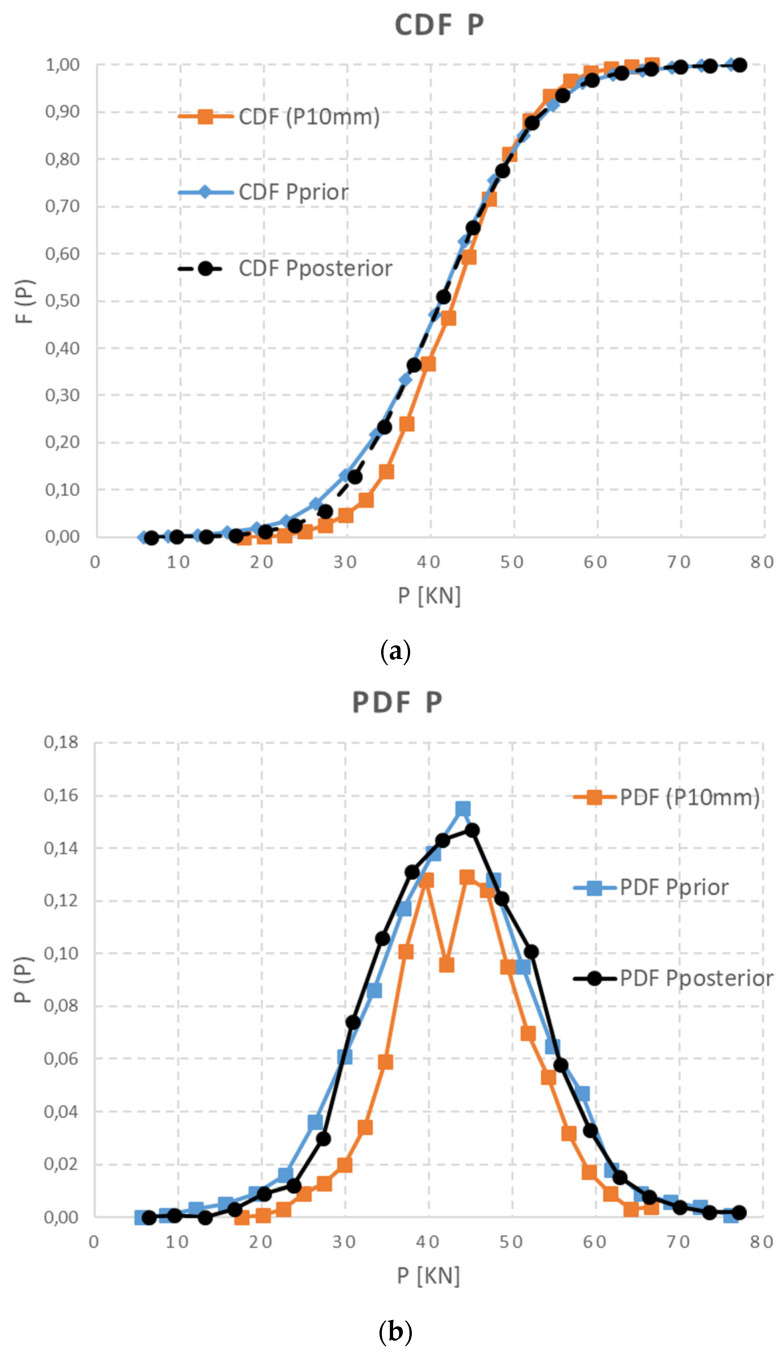
Δσ_c,10_—(**a**) CDF of data set and (**b**) PDF of data set.

**Figure 13:**
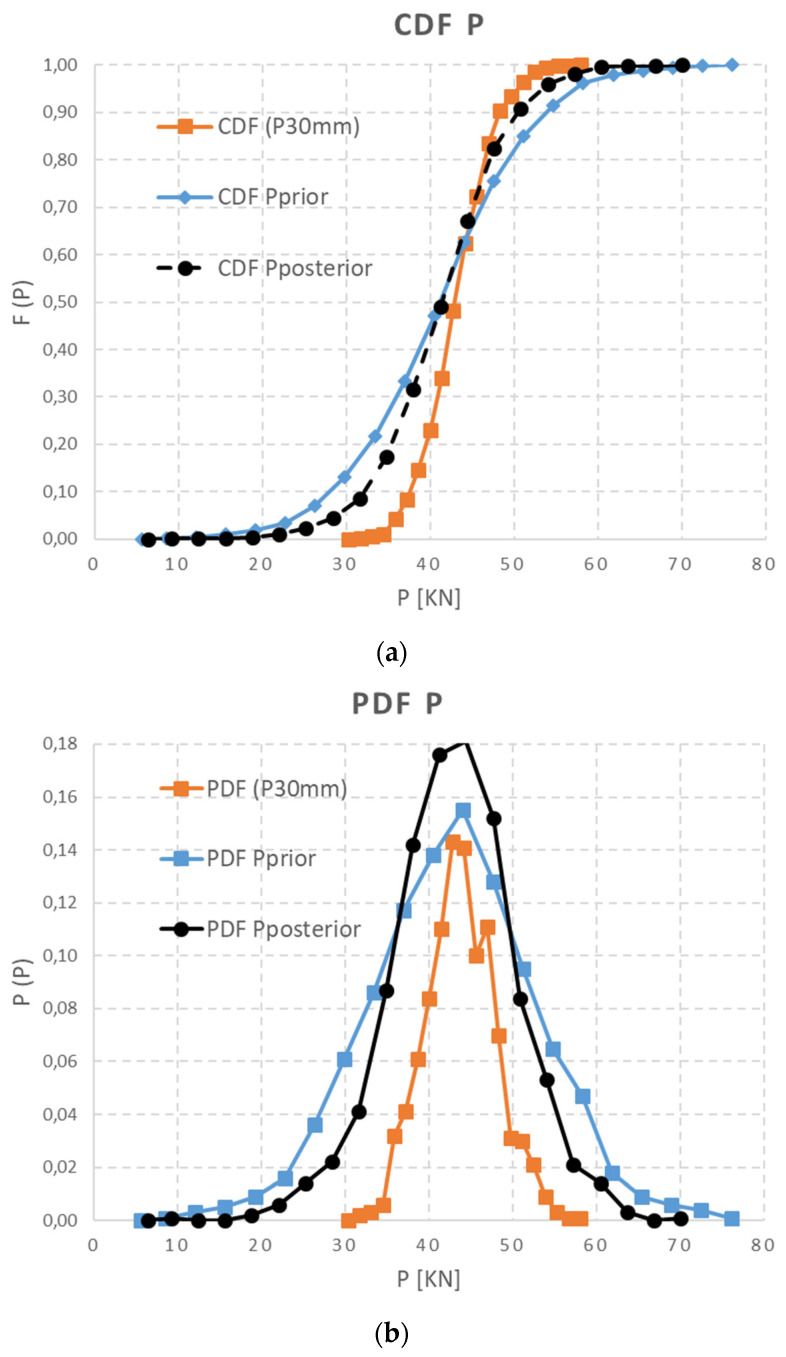
Δσ_c,30_—(**a**) CDF of data set and (**b**) PDF of data set.

The primary predictive data set of calculated prestressing force {P_calc_} can be set as a joint prior distribution function f′ (μ, σ), according to Equation (4), or prior information. The random vector {P (imm)} derived from normal stress releasing, determined given the depth of a saw-cut, can be used in the Bayesian theorem as a likelihood or conditional information. This function is based on a measured data set which specifies and moves assumed calculation. The resulting {P_post_ = P} as a final distribution prestressing force can also be signed as a posterior data distribution f″ (μ, σ). These data were derived from Equation (5) using MC simulation and can be graphically interpreted in PD and CD functions of {P_post_}. The resulting shape and parameters of posterior probability distribution f″ (μ, σ) depends on the distribution function of the measured data sets, as in Figure 12 and Figure 13. The final statistics of the {P} probability distribution function regarding the depth of a saw-cut are presented in Table 6.

## 5. Discussion

Our investigation suggests that a relatively small intervention into the prestressed member can cause sufficient local normal stress relief. Intervention in the form of a maximum 30 mm deep saw-cut is insignificant compared to the dimensions of the cross-section of the prestressed member; the global structural integrity is not affected. Moreover, the saw-cut method can be performed without the application of an external load. Absence of additional external load leads to easier residual prestressing force derivation from the obtained results.

Undoubtedly, prestressing force is the decisive factor in the assessment of existing prestressed concrete structures. However, it is very difficult to obtain its exact value at the time of testing. In addition to the standard analytical evaluation that should be used in all cases, some experimental methods have been verified and applied worldwide [17,18,20,21,26,27]. Nevertheless, these methods provide different results compared to the analytically calculated value. The reason for this is the wide range of factors that affect prestress losses, including the creep and shrinkage of concrete and steel relaxation. It is also possible that the corrosion effect or issues related to inadequate concrete or duct grout quality could influence the analysis.

Generally, Monte Carlo simulation is known as a technique that constructs probability distributions for the possible outcomes of decisions. In the presented study, the MCS was applied to the generation of random variable vectors which were normally distributed. The Bayesian concept can more precisely define the estimated residual value of prestressing force at a certain time P_m (t)_. Usually, the evaluation can only take analytically derived prestress losses into account using the standard approach, as in Eurocode 2 [25], which is similar to the presented study. However, standard calculation of residual prestressing force value is not often sufficient or adequate to deal with such important parameters for global structure reliability evaluation.

## 6. Conclusions

In our paper, there is an obvious coincidence between the prior {P_calc_} and posterior {P_post_} probability distribution functions, with only a 95% confidence level. The reason for such close agreement of both probability functions is that the sleeper specimens were in a very good state after being stored for one year in a covered warehouse without any service or deterioration factors that could affect their structural condition. Moreover, they were kept in a relatively stable environment which inevitably affected the volumetric changes in the concrete. This is why prior and posterior functions are in such good agreement. The kurtosis of posterior functions was higher in both cases, especially for the best-fitted curve for P_post_ (30 mm), due to the strong consistency of the experimental results that form likelihood function. Naturally, if the shape, distribution and displacement of the predictive function of prestress losses on the x-axis were distant from the likelihood function, the posterior probability distribution function would be different.

Unquestionably, it is very important to present the results of methods for determining the state of prestressing in statistical form. This makes it possible to define the probability of the obtained state of prestressing, which is a crucial aspect of the evaluation of the load-carrying capacity of the existing prestressed concrete structure. The implementation of the probability approach to determine prestress losses can be an effective tool, since the evaluation process of existing prestressed concrete structures considers model uncertainties and any possible deteriorations.

## Figures and Tables

**Table 1:** Characteristics of analyzed sleeper.

A_c_ (m^2^)	I_c_ (m^4^)	z_bott_ (mm)	z_upp_ (mm)	ϕ_w_ (mm)	No. of ϕ_w_ (-)	f_pk_ (MPa)	f_p0.1k_ (MPa)	E_p_ (GPa)
0.0324	0.0000816	82	93	7	8	1740	1560	195

**Table 2:** Statistical parameters for P_calc_.

Mean m’ (kN)	Median (kN)	St. Dev. s’ (kN)	Kurtosis (-)	Skewness (-)	Min. (kN)	Max. (kN)	Conf (95) (kN)
40.96	41.04	10.02	0.3586	–0.0036	5.60	75.59	0.6218

**Table 3:** Normal stress relief—saw-cut method.

Sleeper No.	SG1—Δσ_c,i_ (MPa)			SG2—Δσ_c,i_ (MPa)		
	Δσ_c,10_	Δσ_c,20_	Δσ_c,30_	Δσ_c,10_	Δσ_c,20_	Δσ_c,30_
1	2.14	5.78	10.32	1.31	4.92	9.44
2	1.56	4.80	9.16	1.90	4.89	9.68
3	1.48	4.11	8.02	1.49	4.24	8.16
4	1.37	4.72	9.38	1.50	4.74	9.28
5	1.31	4.39	8.37	1.22	4.45	9.55
6	1.42	4.74	8.73	1.21	4.26	8.60

**Table 4:** Cross-sectional parameters.

	A_c_ (m2)	I_c_ (m4)	z_bott_ (mm)	z_upp_ (mm)	e_p,bott_ (mm)	e_p,upp_ (mm)	M_G,o_ (kNm)
Mean	0.0324	0.0000816	82	93	43	59	0.798
St. Dev.	1.619 × 10^−3^	4.082 × 10^−6^	4	4	2	3	0.040

**Table 5:** Statistical parameters for P (imm).

Statistics	Mean m’’ (kN)	Median (kN)	St. Dev. s’’ (kN)	Kurtosis (-)	Skewness (-)	Min. (kN)	Max. (kN)	Conf (95) (kN)
P (10 mm)	43.20	43.21	7.80	0.0587	0.0871	19.11	73.61	0.4839
P (30 mm)	43.17	43.15	3.98	0.1932	0.1220	30.20	57.32	0.2448

**Table 6:** Statistical parameters for P_post_.

Statistics	Mean m″ (kN)	Median (kN)	St. Dev. S″ (kN)	Kurtosis (-)	Skewness (-)	Min. (kN)	Max. (kN)	Conf (95) (kN)
P_post (10 mm)_	41.22	41.38	9.54	0.3869	0.0286	11.39	77.84	0.5920
P_post (30 mm)_	41.34	41.38	7.65	1.6388	0.2352	14.69	79.43	0.4748

## Data Availability

Not applicable.

## Nomenclature

P (A), P (B)	Probability of event A and B.
P (A|B), P (B|A)	Conditional probability.
FORM	First order reliability method.
SORM	Second order reliability method.
JCSS	Joint Committee on Structural Safety.
P_m_ (t)	Prestressing force value in time “t” (kN).
CV	Coefficient of variation.
σ_p,in_	Initial stress in prestressing steel (MPa).
M_G_	Moment due to dead load (kNm).
σ_pm,max_	Maximum stress in prestressing steel according to Eurocode 2 (MPa).
f_pk_	Characteristic tensile strength of prestressing steel (MPa).
f_p,0.1k_	Characteristic 0.1% proof-stress of prestressing steel (MPa).
Δσ_p,r_	Prestress losses due to steel relaxation (MPa).
Δσ_p,r+s+c_	Long-term prestress losses (MPa).
MCS	Monte Carlo simulation.
{P_calc_}	Current random vector of prestressing force.
PDF	Probability density function.
CDF	Cumulative distribution function.
SC	Saw-cut.
SG	Strain gauge.
P_exp,i_	Experimentally determined prestressing force value (kN).
k_i_	Calibration factor of the depth of saw-cuts (-).
Δσ_c,i_	Released normal stress after the application of saw-cuts (MPa).
CS	Cross-sectional function (1/m^2^).
e_p,bott_	Ideal eccentricity of bottom wires from the neutral axis (m).
e_p,upp_	Ideal eccentricity of upper wires from the neutral axis (m).
z_bott_	Position of the neutral axis of an ideal cross-section from the bottom edge (m).
z_upp_	Position of the neutral axis of an ideal cross-section from the upper edge (m).
